# High-Performance Supercapacitor Electrodes from Fully Biomass-Based Polybenzoxazine Aerogels with Porous Carbon Structure

**DOI:** 10.3390/gels10070462

**Published:** 2024-07-15

**Authors:** Thirukumaran Periyasamy, Shakila Parveen Asrafali, Jaewoong Lee

**Affiliations:** Department of Fiber System Engineering, Yeungnam University, 280 Daehak-Ro, Gyeongbuk, Gyeongsan 38541, Republic of Korea; thirukumaran@ynu.ac.kr (T.P.); shakilaparveen@yu.ac.kr (S.P.A.)

**Keywords:** polybenzoxazine, aerogel, porous carbon, electrode material, supercapacitor

## Abstract

In recent years, polybenzoxazine aerogels have emerged as promising materials for various applications. However, their full potential has been hindered by the prevalent use of hazardous solvents during the preparation process, which poses significant environmental and safety concerns. In light of this, there is a pressing need to explore alternative methods that can mitigate these issues and propel the practical utilization of polybenzoxazine aerogels. To address this challenge, a novel approach involving the synthesis of heteroatom self-doped mesoporous carbon from polybenzoxazine has been devised. This process utilizes eugenol, stearyl amine, and formaldehyde to create the polybenzoxazine precursor, which is subsequently treated with ethanol as a safer solvent. Notably, the incorporation of boric acid in this method serves a dual purpose: it not only facilitates microstructural regulation but also reinforces the backbone strength of the material through the formation of intermolecular bridged structures between polybenzoxazine chains. Moreover, this approach allows ambient pressure drying, further enhancing its practicability and environmental friendliness. The resultant carbon materials, designated as ESC-N and ESC-G, exhibit distinct characteristics. ESC-N, derived from calcination, possesses a surface area of 289 m^2^ g^−1^, while ESC-G, derived from the aerogel, boasts a significantly higher surface area of 673 m^2^ g^−1^. Furthermore, ESC-G features a pore size distribution ranging from 5 to 25 nm, rendering it well suited for electrochemical applications such as supercapacitors. In terms of electrochemical performance, ESC-G demonstrates exceptional potential. With a specific capacitance of 151 F g^−1^ at a current density of 0.5 A g^−1^, it exhibits superior energy storage capabilities compared with ESC-N. Additionally, ESC-G displayed a more pronounced rectangular shape in its cyclic voltammogram at a low voltage scanning rate of 20 mV s^−1^, indicative of enhanced electrochemical reversibility. The impedance spectra of both carbon types corroborated these findings, further validating the superior performance of ESC-G. Furthermore, ESC-G exhibits excellent cycling stability, retaining its electrochemical properties even after 5000 continuous charge–discharge cycles. This robustness underscores its suitability for long-term applications in supercapacitors, reaffirming the viability of heteroatom-doped polybenzoxazine aerogels as a sustainable alternative to traditional carbon materials.

## 1. Introduction

Supercapacitors, also called electrochemical capacitors, represent a novel class of energy storage devices acclaimed for their distinctive attributes. Combining the virtues of dielectric capacitors and rechargeable batteries, they excel in swiftly delivering substantial power while surpassing conventional capacitors in energy retention. Their superiority is underscored by a markedly higher power density relative to batteries, primarily attributed to the utilization of carbon materials [1,2,3,4,5]. Carbon’s ascendancy is fueled by its superior performance metrics, ecological compatibility, and cost-effectiveness, with porous carbons emerging as frontrunners in energy storage endeavors. Their expansive surface area, commendable electrical conductivity, and inherent stability render them coveted candidates for electrode material in supercapacitors. Yet, the efficacy of porous carbons is impeded by sluggish kinetics, chiefly stemming from in-pore ion transport hindrance and elongated ion diffusion pathways within the electrode matrix. Consequently, when pore dimensions diminish, the electrode’s potential experiences a notable decline, impeding ion conveyance particularly at elevated current densities, thus severely curbing overall electrode efficiency [6,7,8,9,10]. Addressing these challenges remains imperative for advancing supercapacitor technology towards broader applications and heightened performance thresholds.

Among the myriad materials available, carbon aerogels emerge as a highly promising option, as these materials possess dense, interconnected networks of pores that are both continuous and open. This unique structure endows them with exceptional characteristics, including a large surface area, outstanding electrical conductivity, and high porosity. These attributes make carbon aerogels particularly suitable for supercapacitor applications. However, despite their impressive properties, the production of carbon aerogels remains relatively expensive due to the complexity involved in their synthesis process. Thus, developing cost-effective and straightforward methods for producing multifunctional polymer aerogels remains a crucial area of research [11,12,13,14,15,16].

Polybenzoxazine (PBz), representing an aromatic thermosetting polymer, stands out as a promising material due to its unique properties, such as low water absorption, minimal curing shrinkage, and superior thermal and mechanical characteristics. The synthesis of PBz involves ring-opening polymerization of the benzoxazine (Bz) monomer, which can be initiated through heating or the use of chemical initiators. The Bz monomer itself is derived from a phenolic compound, a primary amine, and formaldehyde, enabling the design of aerogel materials through precise molecular selection and chemical modification. Despite these advancements, the production of PBz aerogels often requires the use of environmentally harmful solvents such as DMF (dimethylformamide) or NMP (N-methyl-2-pyrrolidone) to achieve homogeneous monomer dissolution. The reliance on these solvents raises significant environmental and health concerns, highlighting the need for the development of more sustainable and safer processing methods [17,18,19,20,21].

The ongoing challenge is to optimize these materials and reduce production costs, thereby making supercapacitors a more viable and widespread solution for energy storage needs. Expanding upon prior discussions, this research introduces an innovative method for producing nitrogen-doped carbon aerogel known as ESC-G. This approach, characterized by its simplicity, cost-effectiveness, and efficiency, involves polymerizing a multifunctional benzoxazine monomer. The resulting aerogel exhibits high specific surface area and porosity, making it ideal for various applications. The advantages of both hetero-atom doped carbon and increasing the surface area through gel formation have been utilized in synthesizing ESC-G. Specifically, ESC-G was investigated as an electroactive material for constructing working electrodes tailored for supercapacitors. This study meticulously assessed the electrochemical properties of supercapacitors employing ESC-G compared with those utilizing ESC-N, elucidating the advantages and potential enhancements in performance.

## 2. Results and Discussion

### 2.1. Characterizations of the Synthesized Benzoxazine Monomer

**Figure 1:**
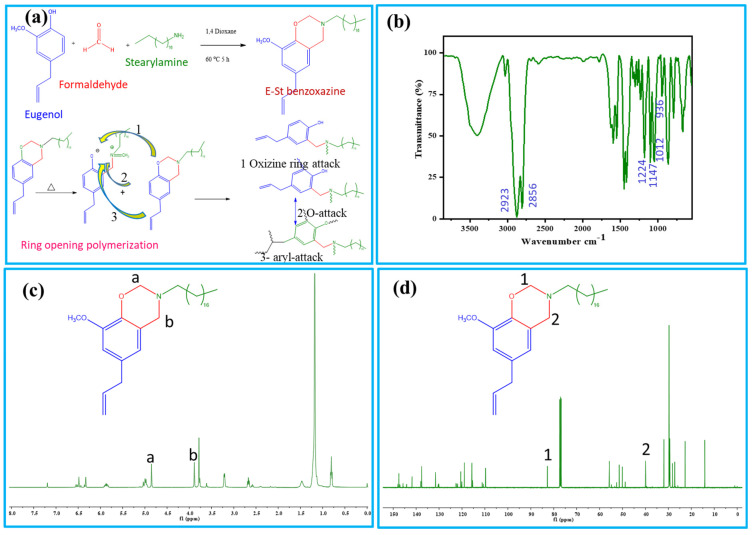
(**a**) Synthesis procedure and ring-opening polymerization of E-St-Bz; (**b**) FT-IR; (**c**) ^1^H-NMR; and (**d**) ^13^C-NMR of E-St-Bz.

**Scheme 1:**
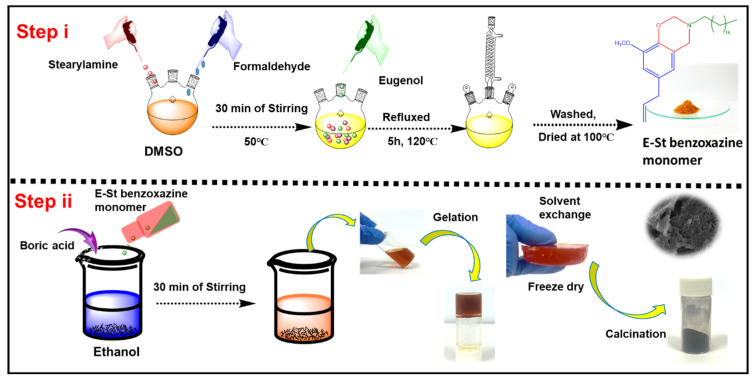
Schematic illustration showing the preparation process of E-St-Bz monomer and PBz- based carbon aerogel.

Schematic illustration of the preparation processes of E-St-Bz and Pbz-based porous carbons are shown in Figure 1a and Scheme 1. The chemical structure of the E-St-Bz benzoxazine monomer was meticulously confirmed using Fourier-transform infrared spectroscopy (FT-IR) and nuclear magnetic resonance (NMR) spectroscopy techniques. In the FT-IR analysis (Figure 1b), distinct peaks revealing the benzoxazine ring structure were identified. Notably, peaks at 1236 and 1028 cm⁻^1^ represented the asymmetric and symmetric stretching of the C-O-C bond, respectively, while the peak at 936 cm⁻^1^ indicated the fusion of a benzene ring with an oxazine ring [22]. Further examination unveiled peaks at 1147 and 1224 cm⁻^1^, corresponding to C-N-C stretching and methoxycarbonyl stretching, respectively. Additionally, intense peaks at 2924 and 2854 cm⁻^1^, denoting C-H stretching vibrations of the alkyl side chain of stearylamine were notable [23,24].

In concurrence with the FT-IR findings, ^1^H-NMR analysis also provided further structural insight, represented in Figure 1c. Peaks at 4.0 and 4.9 ppm were associated with Ar-CH₂-N and O-CH₂-N protons of the oxazine ring, while a singlet at 3.8 ppm confirmed the presence of -OCH₃ protons. Doublets at 3.2, 5.9, and 5.1 ppm were attributed to allyl protons, and multiplets at 2.67, 1.2 and 0.8 ppm indicated the long aliphatic chain attached to the oxazine ring. Furthermore, the presence of the solvent (CDCl₃) was indicated by a peak at 7.2 ppm [25,26]. Subsequent ^13^C-NMR spectra, as shown in Figure 1d, supported these findings, exhibiting characteristic carbon resonances relevant to the oxazine ring, -OCH₃ group, and allyl carbons. In summary, both the FT-IR and NMR spectroscopy techniques provided robust confirmation of the E-St-Bz monomer’s chemical structure, elucidating crucial molecular intricacies essential for understanding and application.

### 2.2. Thermal Behavior of E-St-Bz and Poly(E-St-Bz)

**Figure 2:**
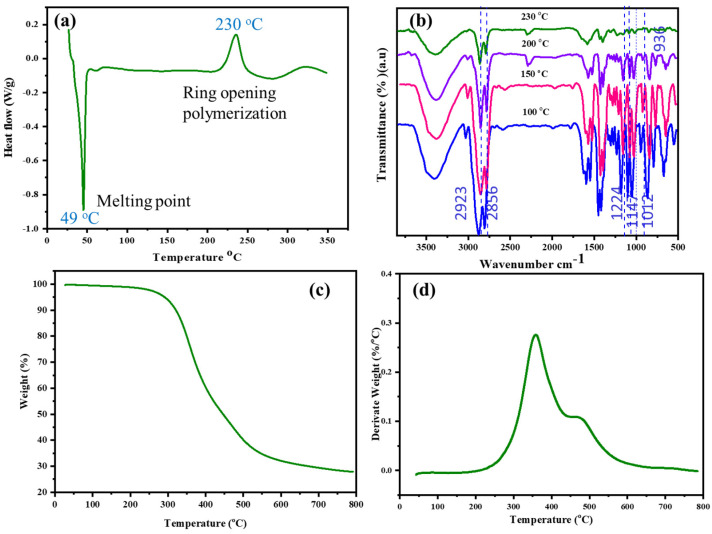
(**a**) DSC curves of E-St-Bz; (**b**) FT-IR spectra of E-St-Bz at different curing temperatures; (**c**) TGA; and (**d**) DTG curve of poly(E-St-Bz).

The polymerization behavior of E-St-Bz was analyzed using differential scanning calorimetry (DSC) under nitrogen, with a heating rate of 10 °C/min from 30 to 350 °C. As observed via the DSC thermogram (Figure 2a), the benzoxazine monomer (E-St-Bz) started to melt at 49 °C, marked by a distinct endothermic peak. The curing process began around 218 °C, with an exothermic peak maximum at 230 °C, indicating the maximum curing process. This curing process between 200–250 °C signifies ring-opening polymerization, a trend that can generally be observed in all sorts of benzoxazine monomers [27,28]. Notably, E-St-Bz exhibited a processing window of 169 °C, indicating favorable processability. Additionally, an exothermic peak at 335 °C was observed, indicating the degradation of aliphatic chains in the E-St-Bz monomer. Confirmation of the curing process of the E-St-Bz monomer was achieved through FT-IR analysis (Figure 2b). FT-IR spectra obtained from the monomer heated incrementally at 100, 150, 200, and 230 °C for 1 h each illustrated the curing progression. Curing involved cleavage of the C-O-C bond within the oxazine ring, resulting in decreases in peak intensity around 936, 1012, 1147, 1224, and 1352 cm⁻^1^, corresponding to stretching vibrations of the oxazine ring (C-O-C) and -CH_2_ of the benzene ring. At higher temperatures (200 °C), involvement of the allyl group within the eugenol moiety in curing was evident, as seen by the disappearance of the peak at 1636 cm⁻^1^, indicative of stretching vibrations associated with the allylic moiety [29,30,31].

Thermogravimetric analysis (TGA) was utilized to evaluate the thermal stability of poly(E-St-Bz), and the results are depicted in Figure 2c,d. This provided essential parameters including initial degradation temperature (Tᵢ), temperatures at 5% (T₅) and 10% (T₁₀) weight loss, and char yield (CY) at 800 °C. Notably, poly(E-St-Bz) exhibited superior thermal stability, displaying Tᵢ = 284 °C, T₅ = 324 °C, and T₁₀ = 353 °C. Furthermore, a char yield of approximately 23.9% was obtained at 800 °C. This thorough analysis sheds light on the complex polymerization behavior and thermal properties of E-St-Bz, hinting at its potential applications across various fields requiring resilient and thermally enduring polymers [32,33].

### 2.3. Structural Analysis of the Prepared Carbon Samples

**Figure 3:**
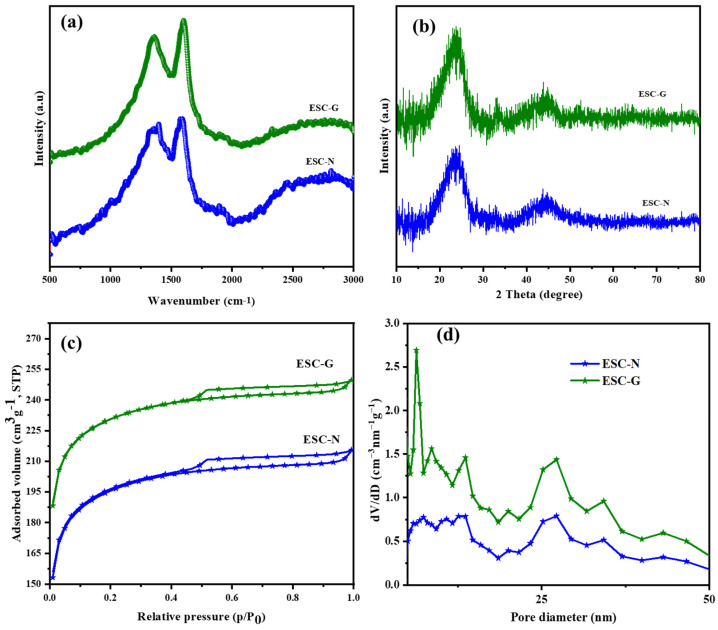
(**a**) Raman spectra; (**b**) XRD patterns; (**c**) BET isotherms; and (**d**) pore size distribution curves of ESC-N and ESC-G.

The benzoxazine monomer underwent two different processes: (i) curing, carbonization, and activation to produce ESC-N; and (ii) gelation, carbonization, and activation to produce ESC-G. To gain a deeper understanding of the structural characteristics and graphitic properties of the synthesized carbon samples, ESC-N and ESC-G, Raman spectroscopy and wide-angle X-ray diffraction (XRD) techniques were employed. The Raman spectra presented in Figure 3a offer valuable insights into the chemical composition and degree of graphitization in the carbon materials. Both ESC-N and ESC-G exhibited two prominent peaks: The D band at 1351 cm⁻^1^ signified the presence of disordered carbon structures, while the G band at 1594 cm⁻^1^ denoted the more ordered, graphitic carbon domains. The intensity ratio of these bands (I_D_/I_G_) served as an indicator of the graphitization level. For ESC-N, a pronounced D band was observed, suggesting a higher concentration of defects within the carbon structure [34]. This could be attributed to the incorporation of nitrogen and oxygen atoms during processing. The calculated I_D_/I_G_ value for ESC-N was found to be 0.96, which is higher compared with the I_D_/I_G_ value of ESC-G (0.85). This signified that the ESC-N possessed a more disordered structure with a higher prevalence of defects compared with the ESC-G. Interestingly, despite the difference in I_D_/I_G_ values for both the samples, they had relatively similar chemical composition and structure, being produced via either the aerogel method or chemical activation. Further details regarding the graphitic domains within the carbon aerogels were obtained through XRD analysis (Figure 3b). Both ESC-N and ESC-G exhibited two broad diffraction peaks at approximately 2θ = 24° and 45°. These peaks corresponded to the (002) and (100) planes of hexagonal graphitic carbon, confirming the presence of graphitic domains within both samples [35].

To quantify the interlayer spacing within these graphitic domains, Bragg’s equation was employed. This equation relates the wavelength of the incident X-rays (λ = 1.5418 Å), the diffraction order (n = 1), the measured angle (θ), and the interlayer spacing (d) within the crystal lattice. Applying this equation revealed a d-spacing (d₀₀₂) of 0.40 nm for each graphite lamella in both the carbons. This value was slightly higher than that observed in conventional graphite (0.33 to 0.34 nm). This enlarged interlayer spacing within the carbon framework could offer a significant advantage for supercapacitor applications [36,37,38,39,40].

Nitrogen sorption measurements were performed on the synthesized samples, ESC-N and ESC-G, to thoroughly investigate their porosity and textural characteristics. These analyses provided crucial insights into specific surface area, pore size distribution (PSD), and pore volume, all of which are key factors influencing the electrochemical performance of supercapacitor electrodes. The N_2_ physisorption isotherms, shown in Figure 3c, included a combined pattern of type I and type IV isotherms for both ESC-N and ESC-G. The type I isotherm, indicative of microporous materials, displayed a sharp rise in N_2_ adsorption at low relative pressures (P/P_0_), which in ESC-G suggested a significant presence of micropores, providing numerous adsorption sites for electrolyte ions. Additionally, both samples showed hysteresis loops at high relative pressures (P/P_0_ close to 1.0), indicating the presence of mesopores with relatively uniform sizes. These mesopores are essential pathways for electrolyte transport within the electrode structure [41,42,43].

Figure 3d further reveals the pore size distribution (PSD) of ESC-N and ESC-G. Both samples exhibited pore diameters less than 2 nm and between 2 to 50 nm, indicating the presence of microporous and mesoporous materials. However, a distinct difference was observed between the two: ESC-N obtained via calcination had a broad mesopore distribution, whereas ESC-G derived from the aerogel featured a narrower mesopore distribution along with a significantly higher pore volume. This specific mesopore structure in ESC-G was attributed to the unique properties obtained through the aerogel process. Moreover, the specific surface area obtained from BET was found to be 289 m^2^ g^−1^ for ESC-N and 673 m^2^ g^−1^ for ESC-G. This substantial difference underscores the effectiveness of the aerogel approach in creating a highly porous material, along with increased surface area [44].

**Figure 4:**
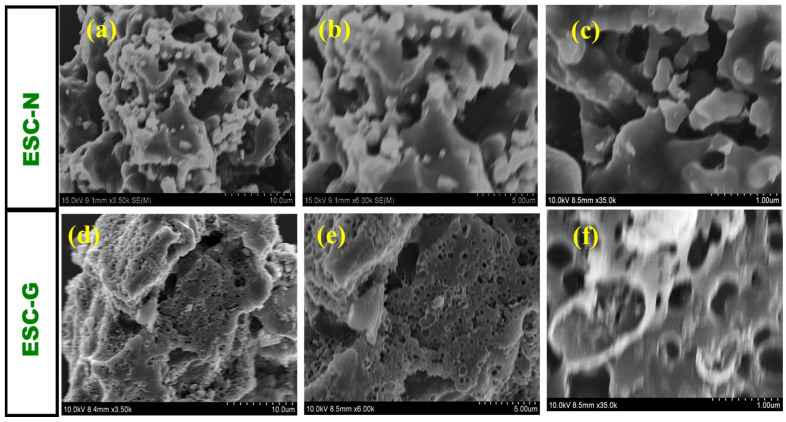
SEM images of (**a**–**c**) ESC-N and (**d**–**f**) ESC-G at different magnifications.

Scanning electron microscopy (SEM) revealed intricate details about the morphology of the synthesized carbon materials, ESC-N and ESC-G. Unlike an ideal supercapacitor electrode, ESC-N features an irregular microparticle structure. These particles, which vary in size from several hundred nanometers to a few micrometers, aggregate to form bulky, porous clusters. Interestingly, these clusters displayed a dual nature, exhibiting both sheet-like and three-dimensional bulk characteristics. The SEM images in Figure 4a–c highlight the disparity between the largest and smallest particles, emphasizing this non-uniformity. Notably, ESC-N exhibited a complete absence of pores, presenting a rough, unbroken surface without visible cavities or macropores. This lack of porosity suggests a limited surface area, which could potentially hinder its performance in supercapacitor applications.

In contrast, the surface morphology of ESC-G, derived from the activation of carbon aerogel, underwent a remarkable transformation. SEM images revealed numerous micropores and mesopores embedded on the surface of the carbon, along with very few voids in the macropore range with a pore diameter of 0.5 mm (Figure 4d–f). These macropores are likely to have been formed by the expulsion of residual solvent during the gelation process. Such extensive porosity is highly advantageous for supercapacitors, as it significantly increases the surface area, offering more sites for electrolyte ion interaction and enhancing capacitance.

**Figure 5:**
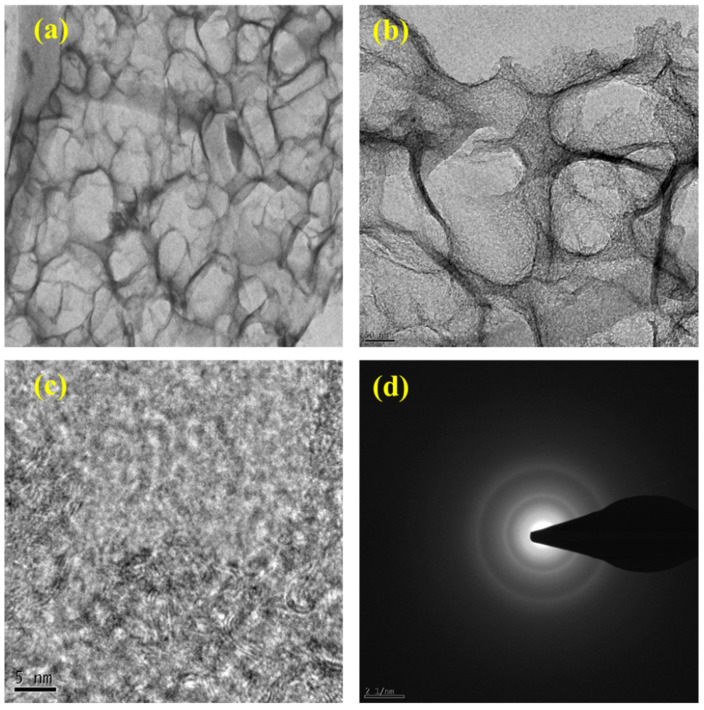
(**a**–**c**) TEM images at different magnifications; and (**d**) SAED pattern of ESC-G.

Transmission electron microscopy (TEM) provided a deeper, high-resolution view of the internal morphology of ESC-G. The TEM images of ESC-G (Figure 5a–d) revealed an open-pore network, a crucial feature for efficient supercapacitor performance. This nano-architecture offers several benefits: it shortens the diffusion pathways for ions, enabling their rapid movement within the electrode, and provides a continuous electron pathway, improving electrical conductivity. Additionally, ESC-G displayed a higher abundance of pores arranged in a more orderly manner. This suggests the presence of sp^2^-bonded carbon in ESC-G, which is highly desirable for its superior electrical conductivity.

**Figure 6:**
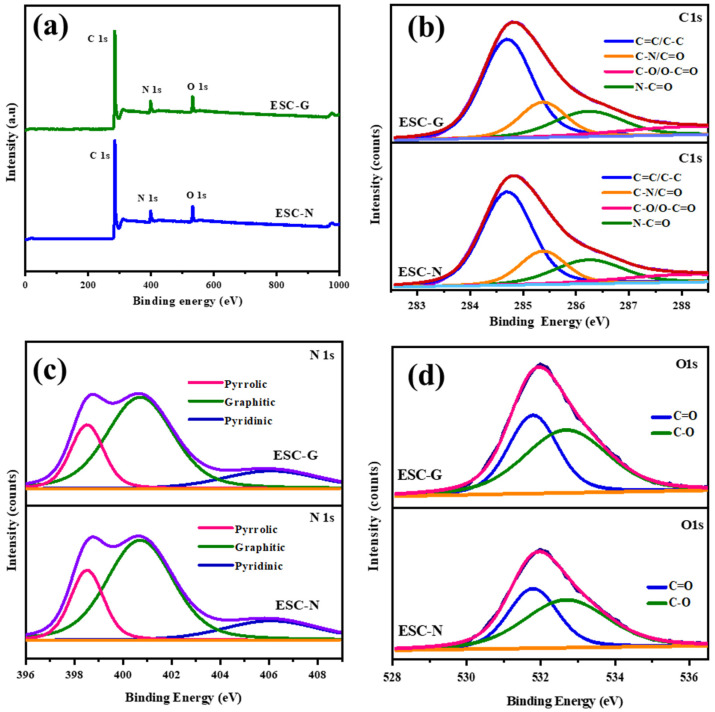
(**a**) XPS survey spectrum; and (**b**–**d**) deconvoluted spectra of ESC-N and ESC-G.

X-ray photoelectron spectroscopy (XPS) was employed as a potent analytical method to delve into the chemical composition and bonding environment of nitrogen and oxygen species present on the surface of the synthesized carbon materials, ESC-N and ESC-G. As shown in Figure 6, the XPS spectra displayed distinct peaks for C 1 s, N 1 s, and O 1 s for both the samples. This confirmed the incorporation of nitrogen and oxygen atoms into the carbon framework, attributable to the use of a benzoxazine monomer as the initial carbon precursor. Additionally, the XPS results verified the absence of impurities in the nitrogen self-doped carbon materials. The XPS survey scan for ESC-N and ESC-G highlighted the characteristic photoelectron peaks for carbon (C), nitrogen (N), and oxygen (O) at binding energies around 281, 401, and 533 eV, respectively [45,46,47]. To gain a deeper understanding of the surface chemistry, deconvolution analyses were performed on the individual C 1 s, N 1 s, and O 1 s peaks, and the results are presented in Figure 6b–d and Table 1.

The deconvoluted C 1 s spectrum shown in Figure 6b revealed four distinct peaks. The first peak, located at 284.7 eV, is attributable to hydrocarbon chains involving C=C and C–C bonds. The second peak at 285.6 eV corresponds to carbon atoms bonded in C–N configurations. The third peak at 286.5 eV signifies the presence of O–C=O, C=N, and C–OH functionalities. The fourth peak at 288.9 eV is indicative of carbon atoms bonded with both oxygen and nitrogen groups (HN–C=O).

Examining the nitrogen environment, the deconvoluted N 1 s spectrum shown in Figure 6c exhibited three distinct peaks, each representing different nitrogen functional groups. The peak at 398.6 eV corresponds to pyrrolic nitrogen, while the peak at 400.8 eV indicates graphitic nitrogen. The third peak at 406.2 eV is associated with pyridine N-oxide, suggesting possible interactions with oxygen-containing species on the surface. The O 1 s spectrum shown in Figure 6d provided additional insights into the oxygen functionalities present on the carbon surface. Deconvolution revealed four peaks at binding energies of 531.3, 533.1, 533.6, and 537.1 eV, corresponding to hydroxyl (C–OH), epoxy (C–O–C), carbonyl (C=O)/carboxyl (COO-), and chemisorbed oxygen or water functional groups, respectively [48,49,50]. This confirmed the presence of oxygen-containing functionalities and possibly entrapped water molecules within the carbon matrix. The XPS analysis unequivocally confirmed the presence of nitrogen and oxygen functionalities within the structure of the synthesized carbon materials.

### 2.4. Electrochemical Characterizations of ESC-N and ESC-G Electrodes

**Figure 7:**
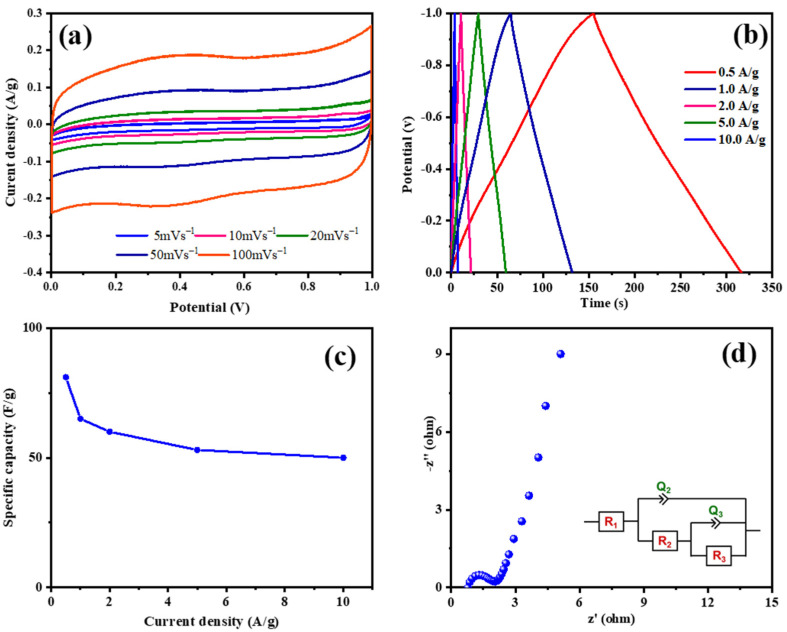
(**a**) CV curves at different scan rates; (**b**) GCD curves at various current densities; (**c**) specific capacitances with changing current densities; and (**d**) an EIS Nyquist plot of the prepared ESC-N electrode.

**Figure 8:**
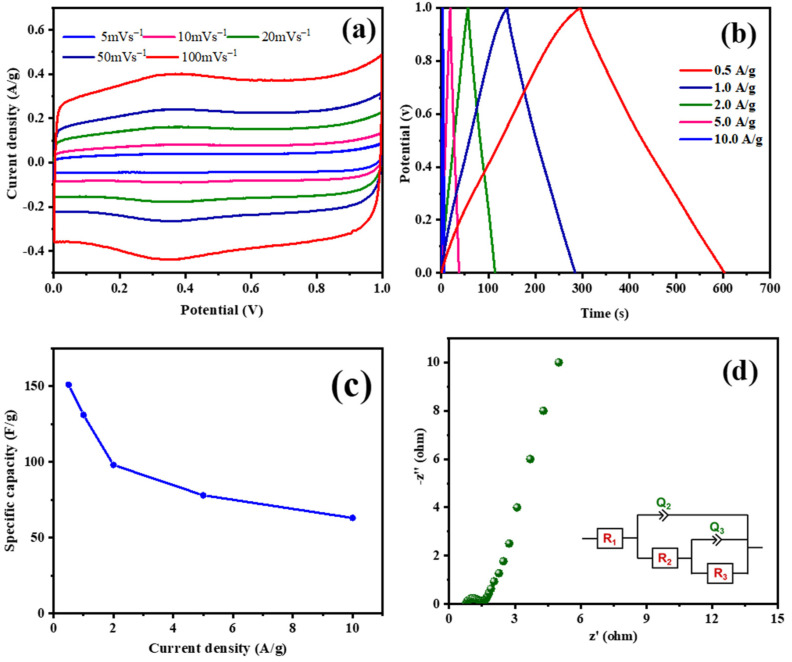
(**a**) CV curves at different scan rates; (**b**) GCD curves at various current densities; (**c**) specific capacitances with changing current densities; and (**d**) an EIS Nyquist plot of the prepared ESC-G material.

ESC-N and ESC-G are nitrogen-enriched porous carbons derived from biomass, exhibiting distinct microstructures that hint at their potential for use as supercapacitor electrode materials. To validate this potential, a comprehensive electrochemical evaluation was conducted using a three-electrode system with 1 M H_2_SO_4_ aqueous electrolyte. A pivotal technique in this assessment was cyclic voltammetry (CV), which gauged the capacitive properties of the electrode materials. Figure 7 and Figure 8 display the electrochemical properties of the ESC-N and ESC-G electrodes. Figure 7a and Figure 8a present the CV curves for ESC-N and ESC-G electrodes across a range of scan rates (5 to 100 mV s^−1^) within a potential window of 0–1 V. Remarkably, even at the highest scan rate of 100 mV s^−1^, the CV curves retained a near-rectangular shape. Moreover, the CV curve area of ESC-G was larger compared with ESC-N. This characteristic suggests an impressive capacitive performance for the ESC-G electrode, indicative of efficient charge storage and release processes.

Galvanostatic charge–discharge (GCD) measurements further elucidated the charge storage and delivery capabilities of supercapacitors. Figure 7b and Figure 8b illustrate the GCD curves for ESC-N and ESC-G electrodes, revealing nearly perfect triangular shapes with minimal IR drop. The slight deviations from perfect triangularity point to some degree of pseudocapacitive behavior. This phenomenon is linked to the presence of nitrogen atoms on the carbon surface, as confirmed by X-ray photoelectron spectroscopy (XPS) analysis [51,52,53]. The presence of nitrogen functionalities enhances pseudocapacitance by creating an electrochemically active interface between the electrolyte ions and the electrode surface. An important performance metric for supercapacitors is specific capacitance, which varies with current density. As depicted in Figure 7c and Figure 8d, the specific capacitance values calculated from the GCD data showed a decline with increasing current density for both the electrodes. Specifically, at current densities ranging from 0.5 to 10 A g^−1^, the specific capacitance decreased from 82 to 52 F g^−1^ for the ESC-N electrode and from 151 to 63 F g^−1^ for the ESC-G electrode. This trend highlights the rate limitations of the electrode material, as higher charging/discharging rates reduce its efficiency.

Electrochemical impedance spectroscopy (EIS) offered additional insights into the interfacial dynamics, diffusion processes, electronic conductivity, and charge transfer resistance within the electrode–electrolyte system. Figure 7d and Figure 8d show the Nyquist plots for the ESC-N and ESC-G electrodes. The plotted intercept on the real axis denotes the solution resistance (R_s_), and the diameter of the semicircle denotes the charge transfer resistance (R_ct_). Notably, ESC-G exhibited a very low R_ct_ value of 0.95 Ω, compared with the ESC-N electrode (R_ct_ = 1.45 Ω), signifying excellent electronic conductivity—a vital trait for high-performance supercapacitor electrodes. This low R_ct_ value significantly contributed to ESC-G’s impressive rate capability.

Obviously, the reduced performance of the ESC-N electrode was attributed to its poorly developed porosity, leading to a lower surface area and uneven nitrogen atom distribution throughout the material. In summary, this study underscores the exceptional potential of ESC-G electrode, a biomass-derived nitrogen-containing porous carbon, as a supercapacitor electrode material. The near-rectangular CV curves, triangular GCD profiles, and low R_s_ and R_ct_ underscore its outstanding capacitive behavior and robust rate capability [54]. This research advances the development of high-performance supercapacitors using sustainable, biomass-derived materials, promoting environmental sustainability while achieving high efficiency in energy storage applications.

### 2.5. Comparison of the Electrochemical Performance of ESC-N and ESC-G Electrodes

**Figure 9:**
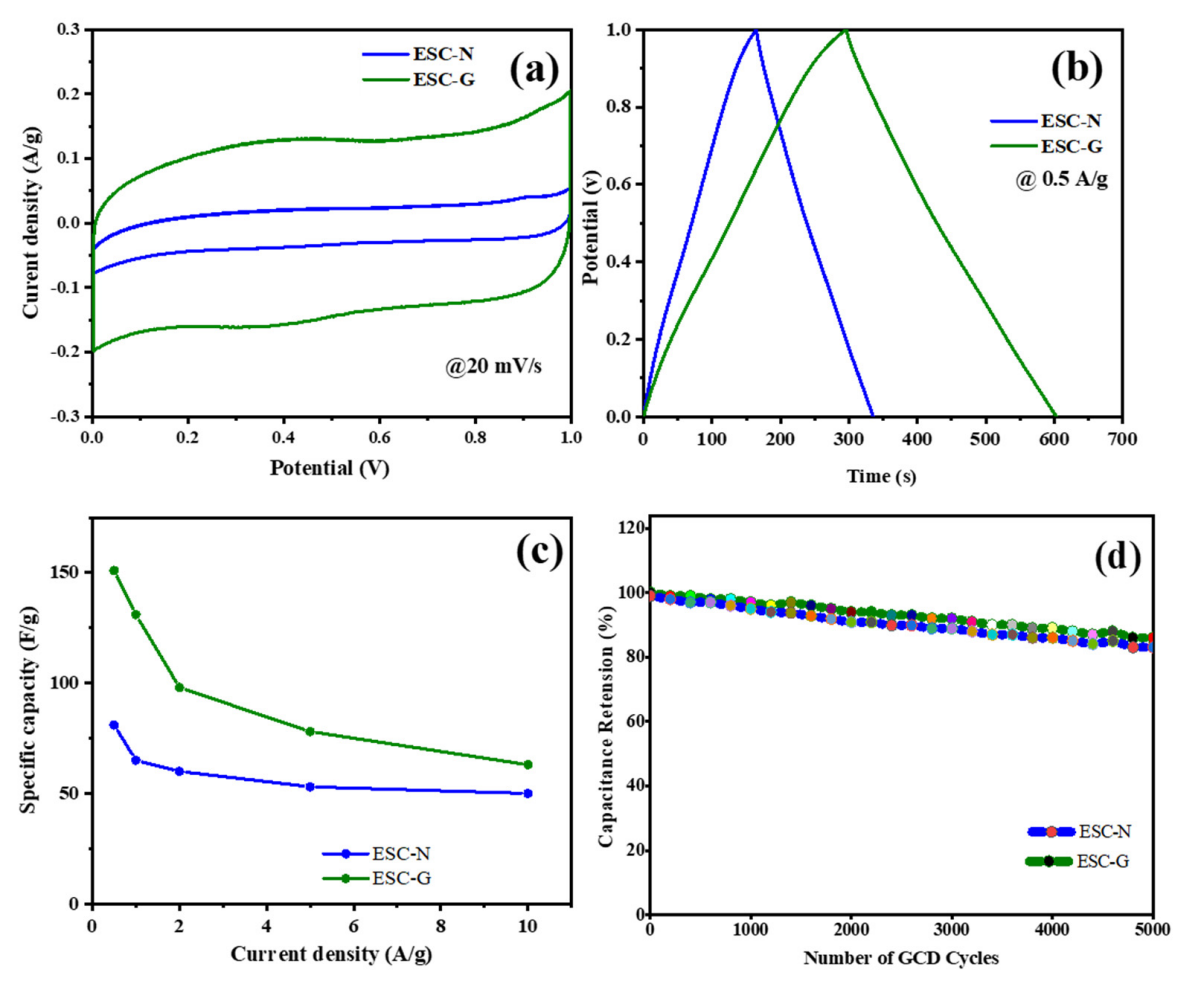
(**a**) CV; (**b**) GCD; (**c**) specific capacitance; and (**d**) cyclic stability of the ESC-N and ESC-G electrodes.

Figure 9a–d provide a comparative visualization of the two electrodes. The CV curve for the ESC-G electrode, evaluated at a scan rate of 20 mV s^−1^, enclosed a noticeably larger area than that of the ESC-N. This indicated a higher capacitance for ESC-G. Similarly, the GCD curves at a current density of 0.5 A g^−1^ revealed a longer discharge time for ESC-G, implying its superior ability to store and release charge. The enhanced performance of ESC-G can be attributed to its unique microporous structure, further complemented by additional micro-, meso-, and macropores. This hierarchical porosity offers numerous advantages. Micropores provide a large surface area essential for efficient ion adsorption, contributing to pseudocapacitance. Meanwhile, meso- and macropores facilitate the smooth infiltration of the electrolyte throughout the electrode, thus maximizing the accessible surface area for ionic interactions. The synergy between pore size and distribution is pivotal in achieving high capacitance [55,56].

Figure 9c quantifies the significant improvement in specific capacitance (C_s_) achieved by incorporating a gel network into the ESC-G electrode. At a current density of 0.5 A g^−1^, the ESC-N electrode exhibited a C_s_ of 82 F g^−1^. Remarkably, this value escalated to 151 F g^−1^ for the ESC-G electrode. This substantial enhancement was directly linked to the optimized porous structure. The interconnected network of pores in ESC-G allows efficient electrolyte penetration, maximizing the electrode surface area available for ion interaction. This effectively meets a key requirement for high capacitance, positioning ESC-G as a promising candidate for supercapacitor applications [57,58,59,60,61,62].

To further explore the practical viability of these electrodes, their cycling stability was evaluated through a GCD study over 5000 charge–discharge cycles (Figure 9d). Notably, the C_s_ of the ESC-G electrode exhibited exceptional stability throughout the test at a current density of 0.5 A g^−1^, although a slight decrease from 151 to 117 F g^−1^ was observed after 5000 cycles and the capacitance retention ratio maintained at 89%. This outstanding stability underscores the long-term durability of ESC-G electrodes, rendering them highly suitable for real-world supercapacitor devices.

## 3. Conclusions

An efficient, adaptable, cost-effective, and highly time-efficient approach was utilized to synthesize heteroatoms containing activated porous carbons, ESC-N and ESC-G, from fully bio-based benzoxazine. This method employed a non-templating technique combined with KOH activation. By utilizing polybenzoxazines as organic precursors, we successfully created a carbon aerogel using ethanol (an ecofriendly solvent), featuring an optimal pore size, making it ideal for electrochemical applications, particularly as an electrode material. This process not only simplifies the synthesis but also enhances the functional properties of the resulting carbon aerogel, broadening its potential applications in various electrochemical devices. The findings from this study clearly demonstrate that the biomass-derived ESC-G electrode, characterized by its hierarchical pore structure and the presence of heteroatoms, offers superior electrochemical performance compared with the ESC-N electrode. The efficient electrolyte penetration and maximized accessible surface area of 673 m^2^ g^−1^ for ESC-G translate into a significant improvement in specific capacitance and cycling stability. These characteristics make ESC-G a promising candidate for the development of high-performance supercapacitors. The advanced structural design of ESC-G, with its well-distributed micropores, mesopores, and macropores, effectively enhances ion transport and storage capabilities. This structural advantage, combined with the presence of heteroatoms, not only boosted the capacitance (C_s_ = 151 F g^−1^) but also ensured remarkable stability (89% capacitance retention) over extended cycling, fulfilling key requirements for practical supercapacitor applications. This research paves the way for further exploration and optimization of biomass-derived materials in energy storage technologies, highlighting the potential of sustainable resources in developing next-generation supercapacitors.

## 4. Materials and Methods

Details relating to the materials, instrumentation methods, synthesis of eugenol-stearylamine-based benzoxazine monomer—E-St-Bz, preparation of porous carbon—ESC-N, preparation of porous carbon via aerogel—ESC-G, fabrication of working electrodes, and electrochemical measurements are given in the Supporting Information.

## Figures and Tables

**Table 1:** Elemental composition of ESC-N and ESC-G.

Type of Atom	Atomic %	
	ESC-N	ESC-G
C	89.35	87.62
N	2.37	3.13
O	8.27	9.25

## Data Availability

The original contributions presented in the study are included in the article/Supplementary Materials, further inquiries can be directed to the corresponding author/s.

## Supplementary Materials

The following supporting information can be downloaded at: https://www.mdpi.com/article/10.3390/gels10070462/s1.
