# Supplementary material for: High-Performance Supercapacitor Electrodes from Fully Biomass-Based Polybenzoxazine Aerogels with Porous Carbon Structure
Source: Gels. 2024 Jul 15;10(7):462. doi: 10.3390/gels10070462 (PMC11275366; doi:10.3390/gels10070462)
Supplement: Supplementary file 1 [file gels-10-00462-s001.zip › gels-3082200-supplementary.pdf]

Supporting Information

High-Performance Supercapacitor Electrodes from Fully Biomass-Based Polybenzoxazine Aerogels with Porous Carbon Structure

Thirukumaran Periyasamy †, Shakila Parveen Asrafali † and Jaewoong Lee *

Department of Fiber System Engineering, Yeungnam University, 280 Daehak-Ro, Gyeongbuk, Gyeongsan 38541, Republic of Korea
* Correspondence: jaewlee@yu.ac.kr
† These authors contributed equally to this work.

Materials and Methods

Materials

Eugenol, paraformaldehyde and stearylamine were acquired from Sigma-Aldrich (USA), while dimethyl sulfoxide (DMSO) and potassium hydroxide (KOH) were obtained from Duksan Chemicals Co., Ltd, Republic of Korea. Hydrochloric acid (HCl), N, N-dimethylformamide (DMF), sodium hydroxide (NaOH) and ethanol were sourced from Daejung Chemicals Co., Ltd., Republic of Korea. Additionally, N-Methyl-2-pyrrolidone (NMP), polyvinylidene fluoride (PVDF), boric acid, and sulfuric acid (H_2SO_4) were procured from Sigma-Aldrich, Republic of Korea. All chemicals were utilized without further purification.

Synthesis of eugenol-stearylamine based benzoxazine monomer E-St-Bz

The synthesis of E-St-Bz benzoxazine monomers commenced with a Mannich reaction between eugenol and stearylamine. In a 500 mL three-necked round bottom flask equipped with a magnetic stirrer, thermometer, and reflux condenser, stearylamine (13.5 g), paraformaldehyde (3 g), and 300 mL of DMSO were successively introduced. The reaction mixture underwent gentle heating to 100 °C and was refluxed with continuous stirring for 1 h. Following this, eugenol (8.2 g) was added drop by drop, and the mixture was stirred at 100 °C for 5 h, resulting in a homogeneous transparent yellow liquid. Upon cooling to room temperature, the mixture underwent transfer into 1 L of 1 N NaOH solution, leading to the precipitation of a substance. The precipitate was isolated via filtration, washed thoroughly with water, and subsequently rinsed with ethanol. After drying at 60 °C in a vacuum oven, the resulting dried sample underwent grinding to yield a pale yellow powder, identified as the E-St benzoxazine precursor. This meticulous process ensures the formation of the desired precursor for E-St-Bz benzoxazine monomers, crucial for subsequent polymerization reactions in material synthesis.

Preparation of polybenzoxazine based carbon aerogels

Scheme 1 outlines the fabrication process and sol-gel transition mechanism of E-St-Bz aerogels. Initially, a solution of boric acid (~1.55 g) in ethanol (50 mL) was prepared to yield the H_3BO_3/EtOH solution (~0.5 M). Subsequently, a specific volume of this solution was combined with E-St-Bz (15 g) in a beaker to form the precursor sol, which was further balanced with additional ethanol and stirred at room temperature for 10 minutes. The resulting precursor sol was then transferred into a Teflon-lined autoclave and subjected to heating at 120 °C for 24 h, followed by 140 °C for 48 h to obtain E-St-Bz alcogels. These alcogels were cooled to room temperature and sequentially immersed in ethanol and n-heptane at room temperature for solvent replacement, with each solvent exchange being refreshed once every 12 h for a total of 3 times. Subsequently, the alcogels were subjected to a CO_2 supercritical drying process.

Following drying, the gel underwent carbonization under a nitrogen atmosphere by heating to 600 °C for 5 h with a ramp rate of 1 °C min^{-1}. The resulting carbonized sample was then thoroughly mixed with twice the amount of KOH powder, and the activation process was carried out at 800 °C for 1 h in a tube furnace under flowing nitrogen with a ramp rate of 3 °C min^{-1}. The resultant products underwent repeated washing with 1 M HCl and deionized water until the pH value of the filtrate reached approximately 7, after which they were dried at 110 °C for 12 h. These processed samples were denoted as ESC-G.

In a parallel procedure, benzoxazine monomer underwent stepwise heating in an oven at 100, 150, 180, 220 and 250 °C for 4 h each. Subsequently, the material was carbonized under a nitrogen atmosphere by heating to 600 °C for 5 h with a ramp rate of 1 °C min^{-1}. The resultant carbonized material was then mixed with an aqueous KOH solution in a weight ratio of 2:1 (KOH : sample), followed by water evaporation at 120 °C. The activation process was conducted at 800 °C for 1 h in a tube furnace under flowing nitrogen with a ramp rate of 3 °C min^{-1}. The products underwent repeated washing with 1 M HCl and deionized water until the pH value of the filtrate approached 7, and were subsequently dried at 110 °C for 12 h. These processed samples were designated as ESC-N. The synthesis process of nitrogen self-doped mesoporous carbon from a multifunctional benzoxazine monomer, as depicted in Scheme 1, involves several crucial steps outlined above.

Instrumentation methods

The ESC-G and ESC-N materials underwent thorough characterization using a suite of physicochemical techniques to elucidate their properties. Field emission scanning electron microscopy (FESEM) coupled with energy-dispersive X-ray spectroscopy (EDS) provided insight into the surface morphology and elemental composition. FESEM with EDS analysis was conducted utilizing a Hitachi S-4800 instrument operating at an accelerating voltage of 4 kV. High-resolution transmittance electron microscopy (HRTEM) images were acquired using an FEI-Tecnai TF-20 transmission electron microscope, operating at an accelerating voltage of 120 kV, to delve into the nanoscale structure of the materials. X-ray diffraction (XRD) measurements were performed on a PANalytical X'Pert3 MRD diffractometer utilizing monochromatized Cu Kα radiation, with scans ranging from 10 to 90° (2θ), shedding light on the crystalline structure and phase composition. Raman spectroscopy, conducted on an XploRA Micro-Raman spectrophotometer (Horiba), explored molecular vibrations within the materials across the spectral range of 50 to 4000 cm^{-1}. Nitrogen adsorption-desorption isotherms were measured at −197 °C using a Micromeritics ASAP 2000 instrument to evaluate surface area, pore size, and pore volumes. Prior to analysis, samples were pre-treated by drying at 120 °C followed by evacuation in flowing argon at 140 °C. Attenuated total reflectance Fourier transform infrared (ATR-FTIR) spectroscopy was performed on a Perkin Elmer Spectrum Two instrument, covering the wavenumber range from 400 to 4000 cm^{-1}, to probe molecular structures and functional groups present in the materials. X-ray photoelectron spectroscopy (XPS) was employed to investigate the elemental composition and chemical states of the surface. Spectra were obtained using a KAlpha instrument (Thermo Scientific) and analyzed using CasaXPS software for high-resolution deconvolution.

Fabrication of working electrode and electrochemical measurements

The fabrication process of the working electrode involved utilizing ESC-G or ESC-N materials in conjunction with polyvinylidene fluoride (PVDF) at a weight ratio of 95:5. Initially, the ESC-G or ESC-N materials were thoroughly ground with PVDF in N-Methyl-2-Pyrrolidone (NMP) to create a homogeneous paste. This paste was then uniformly coated onto a conducting substrate, carbon cloth (CC), with a surface area of 1 cm^2 using the drop-casting method. Subsequently, the electrodes were dried at 100 °C in a hot air oven for 48 h to ensure proper drying.

Post-fabrication, the modified working electrodes underwent evaluation for supercapacitor activity. Various electrochemical measurements, including cyclic voltammetry (CV), galvanostatic charge-discharge (GCD), and electrochemical impedance spectroscopy (EIS), were conducted using a three-electrode system. The testing apparatus employed was the CorrTest-CS350 electrochemical workstation, operating in a 1 M H_2SO_4 aqueous solution.

In this setup, a commercial Ag/AgCl electrode served as the reference electrode, while a platinum plate (1 cm^2) acted as the counter electrode. The working electrodes consisted of ESC-G/CC or ESC-N/CC. During CV measurements, the potential window ranged from 0.0 to 1 V (vs. Hg/Hg_2SO_4) across scan rates of 5 to 100 mV s^{-1}. GCD tests were conducted within a potential window of 0.0 to 1 V (vs. Hg/Hg_2SO_4) at current densities ranging from 0.5 to 10 A g^{-1}. EIS analysis covered frequencies from 0.01 to 100 kHz, employing an alternating current amplitude of 5 mV. All electrochemical assessments took place at room temperature.

To determine specific capacitance (C_s) of the electroactive materials, GCD curves were utilized, employing the formula $C_s = I * \Delta t / \Delta V * m$, where C_s represents specific capacitance, I denotes discharge current (A), Δt signifies discharge time (s), ΔV represents the potential window (V) during discharge, and m stands for the mass (g) of the hydrogel electrode.
